# Complicated systemic JIA with macrophage activation syndrome and pulmonary hypertension responsive to a anti IL-1: case report

**DOI:** 10.1186/1546-0096-12-S1-P227

**Published:** 2014-09-17

**Authors:** Luciana B Paim-Marques, Lidiane Landim, Sarah Firmino, Celia Monteiro

**Affiliations:** 1 Pediatric rheumathology, Infantil Albert Sabin Hospital, Fortaleza, Brazil; 2 Pediatric rheumathology, fortaleza University, Fortaleza, Brazil; 3 Pediatry, Infantil Albert Sabin Hospital, Fortaleza, Brazil

## Introduction

Systemic juvenile idiopathic arthritis (SJIA) is characterized by fevers, rash, and chronic arthritis, and interleukin -1 (IL-1) and IL-6 inhibitors seems to be effective treatments. Pulmonary arterial hypertension (PAH) and macrophage activation syndrome (MAS), which is a unremitting fever, coagulopathy, pancytopenia, and multiple organ dysfunction. These complications can be fatal and may be the result of severe uncontrolled systemic disease activity or influenced by medication exposure.

## Objectives

Describe a 7 year old girl with systemic JIA complicated with macrophage activation syndrome and pulmonary arterial hypertension refractory to numerous treatments, with good response to canakinumab, IL-1 inhibitor.

## Methods

Female patient was diagnosed with systemic JIA at seven year old. At eight years, was hospitalized with desease reactivation in use of cyclosporin and oral prednisone. An echocardiogram showed mild pericardial effusion and moderate PAH (69mmHg), ferritin:5.865mg/dL, triglycerides 428mg/dL, fever, splenomegaly and macrophage activity in bone marrow fulfilled the macrophage activation syndrome (MAS) criteria. Methylprednisolone pulse was started and echocardiographic control showed mild improvement. Anti IL-6 therapy (tocilizumab - 8mg/kg) was started every two weeks, total of 2 doses. Initially, there was clinical improvement and decrease in pulmonary artery pressure (PAP) to 38 mmHg. However, one week later, a new echocardiogram showed mild PH (44mmHg), new pericardial effusion and acute relapse of disease symptoms (fever, rash, adenophathy) and new MAS (ferritin 30.000mg/dl, triglycerides 500mg/dL). The patient underwent a new pulse methylprednisolone and oral prednisolone resulting in clinical improvement and after the second dose of tocilizumab, high transaminases levels were observed indicating anti-IL-6 suspension. After one month, she had a new clinical decompensation with signs of heart failure requiring intensive care. Echocardiogram showed PAP of 95mmHg. A new methylprednisolone pulse therapy was prescribed. After clinical stabilization, received the first dose of IL-1 inhibitor. Second dose of Canakimumabe was performed 4 weeks later, and, at this moment PAP was 48mmHg in absence of fever, rash, arthritis and reduced hepatosplenomegaly.

## Results

In the present case, the patient developed MAS and pulmonary hypertension and had important clinical improvement with anti IL-1 theraphy. MAS is thought to be an acquired form of hemophagocytic lymphohistiocytosis, the real incidence in systemic JIA is not known, but studies show that there are histological changes in the bone marrow, even without clinical symptoms in many patients. HP is a rare condition where immunosuppressants and biological agents may be involved. Diagnose is difficult, but we should have protocols to search it.

## Conclusion

Systemic JIA can be complicated, among others, by MAS and pulmonary arterial hypertension (PAH). Further prospective studies are needed to determine the real factors associated with the development of pulmonary complications.

## Disclosure of interest

L Paim-Marques Paid Instructor for Novartis, Shire, L Landim: None declared, S Firmino: None declared, C Monteiro: None declared.

